# Urological complications of COVID-19: a systematic review

**DOI:** 10.1590/S1677-5538.IBJU.2022.0281

**Published:** 2022-09-24

**Authors:** Luca Schiliró Tristão, Rafael Bresler, Victoria Andrade Modesto, Roni de Carvalho Fernandes, Wanderley Marques Bernardo

**Affiliations:** 1 Faculdade de Ciências Médicas de Santos Departamento de Medicina Baseada em Evidências Santos SP Brasil Departamento de Medicina Baseada em Evidências, Faculdade de Ciências Médicas de Santos (FCMS-UNILUS), Santos, SP, Brasil; 2 Divisão de Urologia Santa Casa de São Paulo São Paulo SP Brasil Divisão de Urologia, Santa Casa de São Paulo, São Paulo, SP, Brasil; 3 Faculdade de Medicina da Universidade de São Paulo Departamento de Medicina Baseada em Evidências São Paulo SP Brasil Departamento de Medicina Baseada em Evidências, Faculdade de Medicina da Universidade de São Paulo, São Paulo, SP, Brasil

**Keywords:** COVID-19, SARS-CoV-2, Infertility, Testosterone

## Abstract

**Purpose:**

COVID-19 continues to be an urgent World issue. Receptors of angiotensin converting enzyme 2 (ACE2), gateway of SARS-CoV-2, are present in the lungs, bladder, prostate, and testicles. Therefore, these organs face high risk of damage caused by the virus and this mechanism may explain non-respiratory symptoms of the disease.

**Materials and Methods:**

This systematic review, guided by the PRIMSA statement, was proposed to elucidate possible urological complications of COVID-19. Searches were carried out in Medline (PubMed), Cochrane (CENTRAL), Embase, MedRxiv and LILACS. Bias analysis was made using the specific Newcastle-Ottawa Scale for each study design.

**Results:**

Search was carried out until April 2022, and 8,477 articles were identified. Forty-nine of them were included in this systematic review. There is evidence that lower urinary tract symptoms and acute scrotum may be signs of COVID-19 in men, although in a small proportion. Also, the disease may have a transitory impact on male fertility, evidenced by several alterations in sperm counts. However, it must be clarified whether this impact is transitory, or may last for longer periods. Several patients showed reduction of total value of testosterone. Two authors linked low levels of testosterone with worse outcomes of COVID-19, suggesting that the hormone may be used as an early biomarker of the severity of the disease. Moreover, it is extremely unlikely that SARS-CoV-2 is transmitted by semen.

**Conclusion:**

This systematic review identified possible repercussions of COVID-19 in the urinary as well as in the male reproductive system.

## INTRODUCTION

In December 2019 began, in the Chinese province of Wuhan, the outbreak of COVID-19 caused by a new coronavirus, the SARS-CoV-2, and on March 11th, 2020, the World Health Organization officially declared it a pandemic. Until August 2022, more than two years after the beginning of the outbreak, the virus reached all continents, affecting approximately 586 million and killed 6,4 million people (1). Although several countries have controlled the disease and have high vaccination rates, there are still some countries where immunization has not reached levels high enough to reduce virus circulation. Also, there are concerns regarding new variants and population groups that refuse the vaccine (2-4). Therefore, elucidation of the effects of SARS-CoV-2 is still very important and relevant.

Receptors of angiotensin converting enzyme 2 (ACE2) are the gateway for the entrance of the virus into the cells. The virus uses the ACE2 receptors for entrance and serin-protease TMPRSS2 receptors for priming of spike protein, similarly to what is observed in SARS-CoV (5, 6). Besides pneumocytes type II, RNA sequencing showed that these receptors are also expressed at myocardial, esophageal, kidney proximal contorted tubules, and urothelium bladder cells (7), and at testicles (spermatogonia, Leydig and Sertoli cells) (8), cholangiocytes (9), ileum and colon enterocytes (10), suggesting that these organs are potentially damaged by SARS-CoV-2, and that this mechanism may explain non-respiratory symptoms caused by the virus. Furthermore, in 2002, during the outbreak of severe acute respiratory syndrome (SARS) it was observed that orchitis is one of the complications of SARS (11). This may be one complication of COVID-19 since SARS-CoV, and SARS-CoV-2 have 79.5% genetic similarity (12) and bind similarly to ACE2 receptors (5, 6).

Several authors have emphasized the need of urological monitoring of COVID-19 patients, not only during the disease, but also to long term complications (13–15). Therefore, a systematic review would be crucial to synthetize the major urologic aspects of SARS-CoV-2. New symptoms of the disease may be detected, expanding alert signs, helping doctors to diagnose better COVID-19, and predicting patients at risk to develop the most aggressive forms of the disease. Once the consequences to urinary and urologic systems are identified, medical decisions may be based on stronger evidence than on the ones currently used.

## OBJECTIVE

The objective of this systematic review was to identify possible urological consequences or complications of patients that were infected by SARS-CoV-2.

## METHODOLOGY

This systematic review was conducted according to the PRISMA statement (16), a recommendation that consists of a checklist and flow diagram to help researchers to improve the report of their systematic reviews and was registered at PROSPERO (17) under the register CRD42020206155. Systematic review of the literature was done using the database Medline (PubMed), Cochrane (CENTRAL), Embase and LILACS. The following search terms were used: (COVID-19 OR COVID19 OR Coronavirus OR 2019-nCOV OR SARS-CoV-2) AND (Urology OR Urological OR Urologic OR Urogenital OR Genitourinary OR Epididymis OR Penis OR Penile OR Prostate OR Scrotum OR Testis OR Testes OR Testicles OR Testicular OR Orchi* OR Leydig OR Sertoli OR Sperm OR Seminal OR Semen).

Two authors evaluated independently the titles and abstracts of the studies, and those meeting the inclusion criteria were selected for this review. In case of disagreements, a third author was consulted.

Articles were selected according to the following eligibility criteria: (I) COVID-19 effects on the urological system; (II) full articles, without language restrictions; (III) articles with relevant outcomes for this review.

Two authors have done the bias analysis using the Newcastle-Ottawa Scale specific to each study design.

## RESULTS

**Figure 1:**
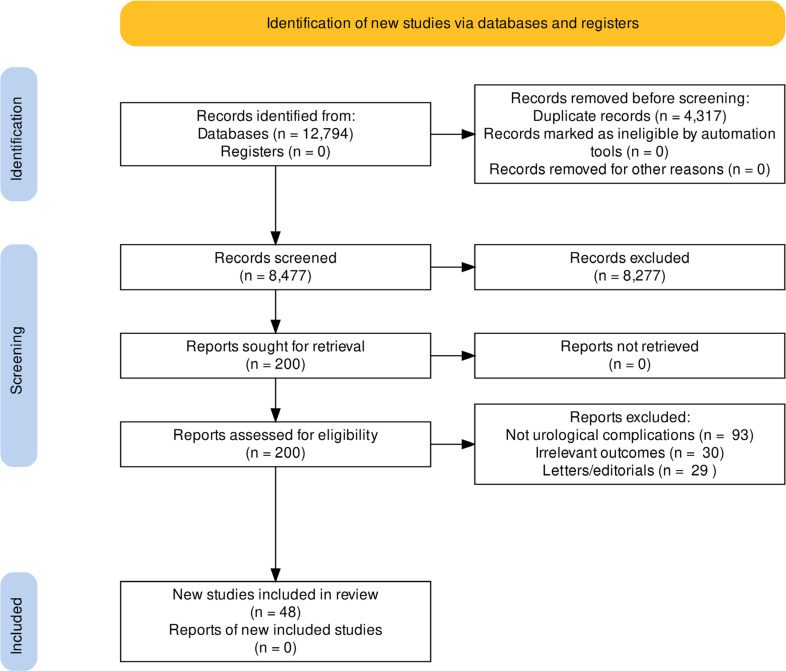
Identification of new studies via databases and registers.

Search was carried out until April 2022 and retrieved a total of 12,794 articles from the scientific databases (Medline: 3,957; Embase: 4,896; LILACS: 356; CENTRAL Cochrane: 3,585). After removal of duplicates, 8,477 titles and abstracts were evaluated and 201 were selected to full reading. According to eligibility criteria, 49 articles (19-68) involving 3,008 infected patients with SARS-CoV-2 were included in this systematic review (Figure-1). Study characteristics are summarized in Table-1.

**Table 1:** Included articles characteristics.

Study	Study design	Location	N	Age (years)	Severity	Study	Study design	Location	N	Age (years)	Severity
Achua et al. (2021) (19)	Cohort	Miami, FL, USA	6	56 (20–87)	Autopsy	La Marca et al. (2020) (43)	Case report	Modena, Italy	1	43	Death
Addar et al. (2020) (20)	Case report	Riad, Saudi Arabia	1	62	Severe	Lam et al. (2020) (44)	Case report	Pembrokeshire, United Kingdom	1	67	Death
Alkhatatbeh et al. (2020) (21)	Cohort	Zarqa, Jordan	253	43 (1–78)	Critical: 12; Severe: 48; Mild: 152; Asymptomatic: 53	Lamamri et al. (2021) (45)	Case report	Le Chesnay, France	1	62	Severe
Best et al. (2021) (22)	Cohort	Miami, FL, USA	30	40	Hospitalized: 8 Non-hospitalized: 22	Lamb et al. (2020) (46)	Cohort	Royal Oak, MI, USA	4	68 (51–85)	–
Bridwell et al. (2020) (23)	Case report	San Antonio, TX, USA	1	37	Mild	Li et al. (2020) (47)	Cohort	Beijing, China	38	–	Acute phase: 23 Recovered: 15
Burke et al. (2021) (24)	Cohort	Orlando, FL, USA	18	32 (24–57)	Moderate: 16; Mild: 2	Li H et al. (2020) (48)	Case series Cross sectional	Wuhan, China	23	40,8 ± 8,5	Ordinary: 14; Mild: 9
Can et al. (2021) (25)	Cohort	Istanbul, Turkey	94	57.5±16.6	> 3 weeks hospitalized	Ma et al. (2020) (49)	Cohort	Wuhan, China	119	39 (35–44)	Critical: 2; Severe: 14; Moderate: 100; Mild: 3
Carreño et al. (2021) (26)	Case report	Bogota, Colombia	1	39	Death	Machado et al. (2021) (50)	Cross sectional	Arkansas, USA	15	23,36 (19–43)	–
Çayan et al. (2020) (27)	Cohort	Mersin, Turkey	46	45,07 ± 18,28	ICU	Mannur et al. (2020) (51)	Case report	Telangana, India	1	36	Mild
			129		Hospital ward	Mumm et al. (2020) (52)	Case series	Munich, Germany	57	62 (59–78)	–
			46		Asymptomatic	Nie et al. (2021) (53)	Case series	Wuhan, China	5	–	–
Chen et al. (2020) (28)	Cohort	Wuhan, China	83	54,2 (38–69)	Non-Severe	Ning et al. (2020) (54)	Case series	Wuhan, China	112	55,5 (23–83)	Severe: 72; Mild: 40
			59	64 (47–78)	Severe	Pan et al. (2020) (55)	Cross sectional	Wuhan, China	34	37	–
Dhar et al. (2020) (29)	Case series	Detroit, MI, USA	39	63,5 (56 - 68,75)	–	Paoli et al. (2020) (56)	Case report	Rome, Italy	1	31	Moderate
Duarte–Neto et al. (2020) (30)	Case series	Sao Paulo, SP, Brazil	5	69 (33-83)	–	Pavone et al. (2020) (57)	Case series	Palermo, Italy	9	42 (31-60)	Mild
Ediz et al. (2020) (31)	Cohort	Istanbul, Turkey	91	38	–	Rastrelli et al. (2020) (58)	Case series	Florence, Italy	4	74,5 (59,5–85,0)	ICU/death
Erbay et al. (2020) (32)	Cohort	Karaman, Turkey	43	31.06 ± 4.2	Moderate				6	72 (33,0–83,5)	Respiratory ICU
			26	30.4 ± 4.8	Mild				21	63 (55,0–66,5)	Hospital ward
Flaifel et al. (2020) (33)	Case series	New York, NY, USA	10	49,5 (22-83)	Death	Rawlings et al. (2020) (59)	Cohort	San Diego, CA, USA	6	(28-45)	–
Fraietta et al. (2021) (34)	Case series	Sao Paulo, SP, Brazil	22	29,5 (23–33)	Mild: 20 Moderate: 2	Ruan et al. (2020) (60)	Case series	Wuhan, China	55	31,15 ± 5,32	All recovered – Severe: 32; Moderate: 31; Mild: 11
Gacci et al. (2021) (35)	Cross sectional	Florence, Italy	43	(18-65)	ICU: 5; Hospital ward: 26; non-Hospitalized: 12	Salonia et al. (2021) (61)	Case-control	Milan, Italy	34	67,0 (59,0–72,0)	Deaths
Gagliardi et al. (2020) (36)	Case report	Forte dei Marmi, Italy	1	14	Mild				51	60,0 (53,0–66,0)	Severe
Guo et al. (2020) (37)	Prospective Cohort	Jinan, China	23	41,04 ± 11,56	Moderate: 5; Mild: 18				174	57,0 (49–65,5)	Moderate
Holtmann et al. (2020) (38)	Cohort	Dusseldorf, Germany	4	40,8 ± 8,7	Moderate				27	49,0 (45,0–55,0)	Mild
			14	42,7 ± 10,4	Mild	Silverman et al. (2021) (62)	Case report	Dayton, OH, USA	1	68	Severe
Kadihasanoglu et al. (2020) (39)	Cohort	Istanbul, Turkey	12	49,9 ± 12,5	Severe	Song et al. (2020) (63)	Case series	Nanjing, China	13	(22–67)	–
			30		Moderate	Temiz et al. (2020) (64)	Cross sectional	Istanbul, Turkey	30	37,21 ± 8,59	–
			47		Mild	Xu et al. (2020) (65)	Cohort	Wuhan, China	39	60 (46,5–65,5)	Severe: 19; Moderate: 20
Kaya et al. (2020) (40)	Cohort	Eskisehir, Turkey	19	38,9 ± 13	Hospitalized	Yang et al. (2020) (66)	Case series	Wuhan, China	12	65 (42-87)	Severe: 3; Moderate: 5; Mild: 2
Kayaaslan et al. (2020) (41)	Cohort	Ankara, Turkey	16	33,5 (18–54)	Moderate: 5; Mild: 11	Zhu et al. (2020) (67)	Case report	Tianmen, China	1	30	Severe
Kim et al. (2020) (42)	Case report	Boston, MA, USA	1	42	Mild						

### Lower urinary tract symptoms

Lower urinary tract symptoms (LUTS) were reported in 5 studies (25, 29, 40, 46, 52). Mumm et al. (52) in a series of 57 cases, reported that 7 patients showed increase of urinary frequency, with a medium of 13.7 urinations at the day of admittance and 11.6 at the 5th day. Other two series, Dhar et al. (29) and Lamb et al. (46), also reported increased frequency in 39 and 4 patients, respectively. Both also verified that patients reported nocturia. Dhar et al. (29) related that 85% of patients presented 13 or more urinations per day and 87% at least 4 urinations at night. Also, Lamb et al. (46) reported urgency and urinary incontinence in 4 patients.

International Prostate Symptom Score (IPSS) was applied in 113 patients in two studies. Kaya et al. (40) did not find significant score differences among previous, during and at hospitalization due to COVID-19. This result is similar to that of Can et al. (25) in patients under 50 years of age (n=32). However, in patients with more than 50 years old (n=62) it was verified an increase of IPSS during hospitalization. Value before COVID-19 was 5.1±4.1 and during infection 9.0±6.4 (p<0. Covid 0001).

### Acute scrotum

Testicle involvement was reported in some of the articles reviewed. Chen et al. (28) studying 142 patients, reported 6 with orchitis, 7 with epididymitis, 19 orchitis-epididymitis and 28 scrotal infections, being the two last more common in patients severely ill (non-severe 3 vs. severe 4; non-severe: 5 vs. severe 10, p<0.05). Ediz et al. (31) (N=91) reported 10 patients with orchitis-epididymitis or testicular pain, and 9 with testicular edema. Pan et al. (55) (N=34) and Holtmann et al. (38) (N=18) reported respectively 6 and 1 case of testicular discomfort. Ning et al. (54) (N=112) did not report any patient with testicular edema, and Alkhatatbeh et al. (21) (N=253) did not report any patient with orchitis. Two case reports reported testicular pain (42,43), one bilateral orchitis (23) and one orchitis-epididymitis in a 14-year-old teenager (36).

La Marca et al. (43) patient presented initial bilateral testicular pain, that evolved 3 days later to dyspnea and death after one week of hospitalization. All other patients presented manifestations of acute scrotum as their initial presentation.

### Autopsies

In this review, it was included 6 autopsies of patients that died due to complications of SARS-CoV-2 infection (19, 30, 33, 48, 53, 66). Yang et al. (66) reported that in 12 patients the medium of quantity of Leydig cells was inferior to control patients (p<0.01). In 9 patients, tubular lesion was described (4 with severe lesion, 5 moderate and 2 mild). Moreover, alterations of spermatogenesis were found in 8 patients. Nie et al. (53) performed autopsies in 5 patients, finding alterations which suggested a disfunction or reduction of Leydig cells and impaired spermatogenesis and alteration of motility of sperms. Li et al. (48) compared 6 patients with COVID-19 with 6 controls and found more apoptotic testicular cells in those infected (p=0.018). In addition, interstitial edema and congestion of testicles and epididymis were reported. Duarte-Neto et al. (30) described two patients with orchitis, Flaifel et al. (33) multifocal testicular microthrombus in 2 patients and Achua et al. (19) reported that 3 patients had spermatogenesis alterations.

### Spermatic parameters

Several alterations such as azoospermia, oligozoospermia, criptozoospermia and teratozoospermia were found (35, 48, 51, 67). Gacci et al. (35) reported that 25.6% of patients developed some of these alterations and that concentration of sperm was lower in more severely will patients. All 9 patients (9/23) reported by Li et al. (48) with oligospermia had fathered children previously to COVID-19 through natural pregnancies. What's more, when compared to controls, sperm concentration of patients with Covid-10 were significantly lower (40.6x106/mL in controls (2, 5, 27, 61), 13.8x106/mL (2, 5, 8, 36) in patients with mild COVID-19 and 10.9x106/mL in ordinary cases). Best et al. (22) observed that patients with the disease had sperm concentrations (11.5 vs. 21.5 x 106/mL; p=0.0048) and total sperm count (12.5 vs. 59.2x106/ejaculation; p=0.0024) lower than control group. Holtmann et al. (38) observed that patients with moderate COVID-19 had reduced total sperm count (11.9±13.4x106/ejaculate) and motility (total amount with progressive motility: 2.4±2.7x106/mL; total amount with full motility: 4.7±5.5x106) while patients with mild disease had not significant alterations (total sperm count: 243.7±140.4 x 106/ejaculate; total amount with progressive motility: 125.3±96.4x106; total amount with full motility: 157.1±120.8x106) when compared to controls (total sperm count: 233.1±234.4x106/ejaculate; total amount with full motility: 102.1±102.3x106; total amount with full motility: 124.0±124.9x106). Finally, Erbay et al. (32) found reduction of progressive motility (28.81%±9.7 vs. 20.92%±9.1, p=0.002) and total (48.69%±12.1 vs. 33.41%±12;3, p<0.001) and reduced vitality (62%±7.0 vs. 58.1%±7.1, p=0.03) when compared to sperm analysis before and after the disease in patients with mild COVID-19, however without alteration of sperm concentration. In patients with moderate COVID-19 it was found reduction in all sperm parameters when compared to controls (total volume: 3.34 mL±1.1 vs. 2.74 mL±0.9, p<0.001; concentration: 35.01x106/mL±14.1 vs. 30.63x106/mL vs. 17.2; p=0.008; total count: 114.53x106±93.66 vs. 90.38x106±83.66; p=0.001; progressive motility: 30.16%±12.1 vs. 21.40%±10.1, p<0.001; total motility : 50.74%±13.4 vs. 31,42%±13.3; p<0.001; vitality 64.6±5.6 vs. 57;4±6.8, p=0.001).

### Presence of SARS-CoV-2 in Semen

The semen of 428 patients of 19 articles was analyzed for possible presence of SARS-CoV-2 (22, 24, 34, 35, 37, 38, 41, 47-50, 54–57, 59, 60-62). Patients included had different severity levels of COVID-19 (from asymptomatic to ICU patients) and semen samples were collected in different periods of the disease. Some patients collected at day 1 and others after 109 days of first symptoms. Of all 428 analyzed samples, only 8 presented the virus in the semen (35, 47, 50).

### Priapism

Five authors reported (N=5) patients with COVID-19 and priapism (20, 26, 44, 45, 62). All had severe forms of the disease (shortness of breath and dyspnea (N=5); intubation and mechanical ventilation (N=3), oxygen support via nasal catheter (N=1), and via CPAP (N=1)) and 2 died. Also, all had a risk factor (overweight/obesity, hypertension, dyslipidemia, diabetes) and 4 were older (62-68 years old). In 5, priapism was ischemic. Interventions in 4 patients included aspiration of blood (n=2), and intracavernous administration of phenylephrine (n=2), etilephrine (n=1) or adrenaline (n=1). Of these, one died and the other three reverted priapism recovering from COVID-19. Lam's et al. (44) patient died before any treatment for priapism.

### Hormones

#### Total Testosterone

Salonia et al. (61) reported that 89.8% of patients with COVID-19 had total testosterone lower than normal (<9.2 nmol/L) while only 14.9% in controls (2.5 (1.0-4.7) vs. 10.4 nmL/L (8.1-13.4); p<0.0001). Furthermore, lower levels of total testosterone in more severely ill patients were observed. Using multiple variated analysis made by the author, total testosterone was inversely associated with admission to ICU (OR=0.54; p<0.0001 (IC95%=0.43-0.67)) and death (OR=0.68; p=0.002 (IC95%=0.53-0.86)). Kadihasanoglu et al. (39) also showed that patients with COVID-19 presented lower values of total testosterone than controls (182.52 vs. 332 ng/dL; p<0.0001). Rastrelli et al. (58) showed in 31 patients admitted to respiratory ICU (5.0 nmol/L (1.8-7.6)) or ICU/deaths (1;0 nmol/L (0.2-1.9)) lower levels than normal (considered by the author - <8.6 nmol/L), while hospital ward patients presented normal values (medium) (8.8 (4.1-16.2)). Ma et al. (49) (COVID-19: 3.97 (3.14-5,74)) vs. control: 4.43 ng/mL (3;53-5.24); p=0.1886) and Xu et al. (65) (COVID-19: 3.3932±1.081 vs. control: 3.838 ng/mL±0.96; p<0.05) did not find significant differences among patients with COVID-19 and controls. Lastly, Çayan et al. (27) reported that patients showed a medium of normal values (308 ng/dL (18-931); normality: ≥ 300 ng/mL), but hypogonadism was observed in 113 patients (51.1%). Additionally, in 24 patients that presented the previous values of testosterone (before SARS-CoV-2 infection) a reduction of total values was observed, from 458±198 ng/dL to 315±120 ng/dL (p=0.003).

## FSH

Xu et al. (65) was the only author that found significant differences of values between sick patients and health controls (although at normal range) (1.27-19.26 mLU/mL), with lower levels in patients with COVID-19 (8.763±4.952 vs. 14.407±12.918; p<0.05). Ma et al. (49) and Kadihasanoglu et al. (39) did not find differences between groups. Salonia et al. (61), showed that more severely ill patients had lower levels of FSH, although did not also find any significant differences between patients with or without COVID-19 (mild: 7.0 mU/mL (3.9-8.3); moderate: 6.9 (4.5-9.9); severe: 3.9 (2.6-5.8); deaths: 4.6 (3.8-6.4); p<0.0001). However, Çayan, et al. (27) reported that patients at ICU had higher levels than asymptomatic patients (8.41±4.38 mIU/mL vs. 5.26±2.68; p=0.02).

## LH

A significant difference was found between COVID-19 patients and controls in four articles. In three of them, the patients showed higher values of LH (Ma, et al. (49)): 6.36 mIU/mL (4.63-8.37) vs. 3.38 (2.48-4.52), p<0.0001; Salonia et al. (61): 4.7 mU/mL (3.0-6.7) vs. 4.1 (3.0-5.4), p=0.005; Kadihasanoglu et al. (39): 5.67±4.52 U/L vs. 4.1±2.62, p=0.0001). On the other side, Xu et al. (65) reported that patients with COVID-19 showed lower levels than controls (5.519 mIU/mL±2.705 vs. 8.051±6.048, p<0.05). Rastrelli et al. (58) reported that patients with COVID-19 at ICU or that died presented higher levels than those admitted to hospital wards (11.2 U/L (9.0-19.3) vs. 6.6 (4.6-9.6); p=0.037) and above normality (LH: 1.7-8.6 U/L). On the other side, Çayan et al. (27) did not find any statistically significant differences among asymptomatic patients (5.31±2.38 mIU/mL) in hospital wards (5;73±2.22) and those at ICU (5.97-3.17).

### Prolactin

Kadihasanoglu et al. (39) showed that patients with COVID-19 had higher levels than controls (9.6±5.59 ug/L vs. 7.5±1.86; p=0.0007). But Xu et al. (65) did not find any difference between patients with or without COVID-19 and Çayan et al. (27) did not find significant differences among different levels of severity of the disease.

### Estradiol

Xu et al. (65) reported significant differences between groups (COVID-19: 50.9±18.8 vs. controls: 34.9±18.5; p<0.05) with values above normal (≤47 pg/mL) in patients with COVID-19. Salonia et al. (61) found higher levels in patients with COVID-19 than in controls (35.0 pg/mL (22.4-44.2) vs. 23;3 (19.0-27.9); p<0.05) and, finally, Çayan et al. (27) did not find significant differences among different severities of the disease.

## DISCUSSION

SARS-CoV-2 infection may cause several impacts on male urinary and genital systems. Many important changes in semen parameters in patients (azoospermia, oligozoospermia and criptozoospermia) comparing before and after infection were observed. The presence of the virus in semen was rare and reported in only 8 of the 428 samples analyzed. Patients with COVID-19 showed reduced values of total testosterone, with lower values in more critically ill patients. Furthermore, urinary frequency increase, LUTS, nocturia, urgency and incontinence were reported in several patients. Orchitis, epididymitis, edema, pain, and tenderness of testicles were also reported by several authors, demonstrating a possible testicular impact of COVID-19. Five cases of priapism were also reported. Other findings, such as IPSS alteration and changes in levels of hormones were heterogeneous.

Since the beginning of COVID-19 pandemics, there is great concern about the effects of SARS-CoV-2 on urological system, especially on the male reproductive system, due to the presence of ACE2 and TMPRSS2 receptors and the great similarity of SARS-CoV-2 and SARS-CoV. The presence of these receptors in male genital organs and in urinary bladder may explain a possible direct mechanism of action of this virus in these organs, which may explain several findings such as LUTS, IPSS increase, orchitis, epididymitis and alterations of sperm count.

Another possible explanation for the urological involvement that may occur concurrently with the direct attack of the virus is the damage caused by the inflammatory activity. COVID-19 is considered an inflammatory disease, evidenced by the cytokines storm (68). It is possible that the production of oxygen reactive species may stimulate pathways for cytokine release with exaggerated inflammatory response (69) with several cellular damage. Endothelitis caused by the virus may be one of the multiple mechanisms that cause LUTS and increase of urinary frequency (70). Duarte-Neto et al. (71) suggests that the clearance of viral antigens in the testis take longer than expected and that this can induce severe cellular changes, such as loss of Leydig cells.

In the included articles, it was observed several alterations in patients’ sperm parameters, such as azoospermia, oligozoospermia and criptozoospermia. One important finding was reported by Li et al. (48), that showed that all patients with oligospermia had already fathered children by natural conception, suggesting that the disease may impair, even if transitorily, male fertility, and that the alterations may or may not be present before infection. The physiopathology of COVID-19, in particular the inflammatory response, may damage testicular cells and compromise the quality of sperm. It is also possible that fertility may be affected by several drugs used during treatment of COVID-19, such as antibiotics, corticosteroids, chloroquine, among others (72). Carneiro et al. (73) points out that there are asymptomatic epididymal injuries since they found color Doppler ultrasound changes in 42.5% of patients without symptoms of epididymitis. They hypothesize that these injuries can have deleterious impact on seminal parameters. More studies comparing spermatic parameters before and after the disease are needed to define if infertility may be a complication of COVID-19. It is also important to follow up patients over time to verify if the impact is transitory or if it can be long-lasting.

Evidence synthesis showed that the disease may affect testosterone levels. Three articles showed that patients with COVID-19 had lower values compared to controls, particularly those more severely affected. Rastrelli et al. (58) demonstrated that the values were lower the more severe the disease. Patients with COVID-19 showed values below normal according to authors. This result is extremely important since Salonia et al. (61) univariate analysis showed that total testosterone level was inversely associated with admission to ICU, suggesting that the hormone may be an early biomarker of severity of COVID-19. Such association was also suggested by Rastrelli et al. (58). It is possible that the reduction of testosterone may last until after the recovery of COVID-19, impacting men's sex lives. Teixeira et al. (74) suggest that a decreased testosterone/luteinizing hormone ratio correlated with high levels of C-reactive protein and white blood cell count, reported by some authors, can mean a transient stage of hypogonadism. In an experimental study, Carrasco et al. (75) inoculated in rats nucleocapsid protein, that have high IgG antibodies against it in COVID-19 patients and found low serum levels of testosterone and free testosterone in these rats compared with a control group. They suggest that this hormone imbalance can be linked with a post-COVID-19 syndrome hypogonadism. However, prospective studies are needed to clarify all this hypothesis.

Regarding other hormones, the findings were heterogeneous. The great variation of findings may be due to previous diseases and conditions (before infection by SARS-CoV-2) that could have affected hormonal levels of patients. However, since these studies were observational and without previous knowledge of hormonal levels before the disease, it is not possible to conclude with accuracy the reasons of the findings.

Aside from the possible pathological effects that the disease can cause, sexual transmission of the virus was uncertain at the beginning of the pandemic. This review concluded that transmission through semen is highly unlikely. In only 8 of 428 samples analyzed the virus was found. Paoli et al. (76) suggested that virus detection could have occurred due to contamination of sample during masturbation, a non-sterile way of collecting, different from the nasal swab or venipuncture. There is also the possibility of cough contamination. Massarotti et al. (77) believes that contamination may have occurred due to residual virus from the respiratory tract. There is evidence of the presence of SARS-CoV-2 in urine (78), although in a few patients. Moreover, even if RT-PCR in semen is positive, the result only implies the presence of viral RNA in the sample, and that there may be no viable virus to be transmitted through semen ((Paoli et al. (76)). New studies are needed to determine if it is possible, even in a small proportion of patients, the transmission of SARS-CoV-2 through semen.

The COVID-19 pandemic strongly impacted all medical specialties and urology was no different. Initially, the biggest concern was about the risk of patients undergoing surgery to contract SARS-CoV-2, especially cancer patients. Anjos-Silva et al. (79) reported that the longer the hospital stay after urological surgeries, the greater the risk of contracting COVID-19 and being a fatal case. It was considered that elective urological surgeries can be safe but that urgent cases need special care to avoid contamination. Zampolli et al. (80) demonstrated that robotic and laparoscopic surgeries are safe regarding the risk of infection by SARS-CoV-2 and that the fact that they lead to a shorter hospital stay is a benefit in this situation. Several authors have been suggesting protocols to reduce the risk of infection by the virus. It is agreed that all staff members should wear Protective Personal Equipment, such as protective eyewear and N95 or PFF2 (81) masks. Furthermore, all patients should be considered suspects until proven otherwise and that all healthcare professionals should be tested in case of suspicion (82). Regarding the postponement of surgeries, especially those involving cancer patients, it is extremely important that each case is analyzed individually, considering the patient's condition and preferences and hospital conditions for big surgeries (81, 83). Cancellations and postponements of elective surgeries, medical appointments, diagnostic procedures, and non-emergency surgeries were very common in this period, strongly harming the training of residents in urology. Prezotti et al. (84) analyzed the impact of the pandemic on urology medical residencies through questionnaires. Residents estimate that the median damage to the urological training was 6.0 [3.4 -7.7] in a scale from 0-10. In addition to the impairment in training, there was an important impact on health and quality of life, with several residents reporting weight gain, reduced physical activity, development of depressive symptoms, in addition to increased alcohol consumption and smoking. Faced with this impact on urological practice, one way to work around some of these problems is the implementation of telemedicine. Despite the impossibility of carrying out a physical examination, online consultations were of great importance in this period, reducing the chance of infection by SARS-CoV-2, promoting self-care, and enabling the training of residents (85).

Evidence of the urological involvement in patients infected by SARS-CoV-2 is limited. Bias analysis showed that only 5 articles presented low risk of bias and all others presented moderate or high risk of bias (Table-2). Most studies had low methodological quality, with only a limited number of patients, with heterogeneous characteristics regarding severity of the disease, age, comorbidities and received treatment. Lack of follow-up after COVID-19 is also another limiting factor since most studies were not longitudinal and due to the short period since the beginning of the pandemics. There are no studies that report exam results before and after the disease, limiting the extension of the conclusions of this review. It was not possible to perform a metanalysis due to impossibility to compare studies with different methodologies (study designs) and different measures used. Prospective studies with good methodologic quality, and longer follow-up are needed to determine the real impact of the disease on the male genital and urinary systems. This systematic review summarizes in a single article the main changes that COVID-19 can cause in the urological system. We describe several points that should be further investigated, such as changes in sperm parameters, since it has a potential impact on the reproductive life of men, and pathological findings of the virus attack on the testes. In the discussion, we were able to discuss the findings with several authors, providing urologists with an overview of the involvement of the urological system.

**Table 2:** Bias analysis.

Author	Best et al. (2021) (22)	Burke et al. (2021) (24)	Can et al. (2021) (25)	Çayan et al. (2020) (27)	Erbay et al. (2021) (32)	Guo et al. (2021) (37)	Holtmann et al. (2020) (38)	Kadihasanoglu et al. (2021) (39)	Kaya et al. (2021) (40)	Ma et al. (2021) (49)
Selection										
1	1	1	0	1	1	1	1	1	1	1
2	1	0	0	0	1	0	1	1	0	0
3	1	1	1	1	1	1	1	1	1	1
4	0	0	1	0	1	0	0	0	0	1
Comparability										
1	1	0	0	0	1	0	1	1	0	0
Outcome										
1	1	0	1	1	1	0	1	1	1	1
2	1	0	1	0	1	1	0	0	1	1
3	1	0	1	1	1	1	1	1	1	1
Total	7	2	5	4	8	4	6	6	5	6

Cohort – Newcastle-Ottawa Scale

More information see APPENDIX 1

## CONCLUSION

Although further studies are needed, this systematic review identified possible urological consequences or complications of COVID-19 such as changes of micturition pattern, urological urgencies, autopsies findings, sperm alterations, hormonal changes, and that the sexual transmission is highly unlikely.

## APPENDIX 1:

**Table:** Cross-sectional – Newcastle-Ottawa Scale.

Author		Achua et al. (2021) (19)	Alkhatatbeh et al. (2020) (21)	Chen et al. (2021) (28)	Ediz et al. (2021) (31)	Gacci et al. (2021) (35)	Kayaaslan et al. (2020) (41)	Lamb et al. (2020) (46)	Li et al. (2020) (47)
**Selection**									
	1	0	1	1	1	1	1	0	0
	2	1	0	0	0	0	0	0	0
	3	1	1	1	1	1	1	0	1
**Confounder**									
	1	0	0	0	0	0	0	0	0
**Outcome**									
	1	1	1	1	0	1	1	1	1
**Total**		**3**	**3**	**3**	**2**	**3**	**3**	**1**	**2**

		Li et al. (2020) (48)	Machado et al. (2021) (50)	Pan et al. (2020) (55)	Rastrelli et al. (2021) (58)	Rawlings et al. (2020) (59)	Ruan et al. (2020) (61)	Temiz et al. (2021) (64)	Xu et al. (2021) (65)
**Selection**									
	1	1	1	1	1	0	1	1	1
	2	0	0	0	1	0	0	1	1
	3	1	1	1	1	1	1	1	1
**Confounder**									
	1	0	0	0	0	0	0	2	1
**Outcome**									
	1	1	1	1	1	1	1	1	1
**Total**		**3**	**3**	**3**	**4**	**2**	**3**	**6**	**5**

**Table:** Case report – Newcastle-Ottawa Scale.

Author		Addar et al. (2020) (21)	Bridwell et al. (2021) (23)	Carreño et al. (2021) (26)	Gagliardi et al. (2020) (36)	Kim et al. (2020) (42)	La Marca et al. (2020) (43)	Lamamri et al. (2021) (45)	Mannur et al. (2020) (51)	Paoli et al. (2020) (56)	Silverman et al. (2021) 62)	Zhu et al. (2021)(67)
**Selection**												
	1	1	1	1	1	1	1	1	1	1	1	1
	2	0	0	0	0	0	0	0	0	0	0	0
	3	0	0	0	0	0	0	0	0	0	0	0
	4	0	0	0	0	0	0	0	0	0	0	0
**Confounder**												
	1	0	0	0	0	0	0	0	0	0	0	0
**Exposure**												
	1	1	1	1	1	1	1	1	1	1	1	1
	2	0	0	0	0	0	0	0	0	0	0	0
	3	0	0	0	0	0	0	0	0	0	0	0
**Total**		**2**	**2**	**2**	**2**	**2**	**2**	**2**	**2**	**2**	**2**	**2**

**Table:** Case series – Newcastle-Ottawa Scale.

Author		Dhar et al. (2020) (29)	Flaifel et al. (2021) (33)	Fraietta et al. (2022) (34)	Li et al. (2020) (41)	Mumm et al. (2020) (52)	Nie et al. (2021) (53)	Ning et al. (2020) (54)	Pavone et al. (2020) (57)	Song et al. (2020) (63)	Yang et al. (2020) (66)
**Selection**											
	1	1	1	1	1	1	1	1	1	1	1
	2	0	0	0	0	0	0	1	1	0	1
	3	0	0	0	0	0	0	0	0	0	0
	4	0	0	0	0	0	0	0	0	0	0
**Confounder**											
	1	0	0	0	0	0	0	0	0	0	0
**Exposure**											
	1	0	1	1	1	1	1	0	1	1	1
	2	1	1	1	1	1	1	1	1	1	1
	3	0	0	0	0	0	0	0	0	0	0
**Total**		**2**	**3**	**3**	**3**	**3**	**3**	**3**	**4**	**3**	**3**

**Table:** Case-control – Newcastle-Ottawa Scale.

Author		Salonia et al. (2021) (61)
**Selection**		
	1	1
	2	0
	3	1
	4	1
**Comparability**		
	1	1
**Exposure**		
	1	1
	2	1
	3	1
**Total**		**7**
